# Supplementary material for: Innovative Antifungal and Food Preservation Potential of Eucalyptus citriodora Essential Oil in Combination with Modified Potato Peel Starch
Source: Foods. 2025 Feb 12;14(4):602. doi: 10.3390/foods14040602 (PMC11854413; doi:10.3390/foods14040602)
Supplement: Supplementary file 1 [file foods-14-00602-s001.zip › foods-3458721-supplementary.pdf]

Antifungal and food preservation potential of *Eucalyptus citriodora* essential in combination with modified potato peel starch

Nabila Khan[a], Saeeda Fatima[a], Muhammad Bilal Sadiq[a*]

[a] Kauser Abdullah Malik School of Life Sciences, Forman Christian College (A Chartered University), Lahore, 54600, Pakistan.

***Author for correspondence: Muhammad Bilal Sadiq; Kauser Abdullah Malik School of Life Sciences, Forman Christian College (A Chartered University), Lahore, 54600, Pakistan.**

Tel: +92 (0) 336-0416900

Email: bilalsadiq@fccollege.edu.pk ;m.bilalsadiq@hotmail.com

Supplementary Material

Table S1: Color analysis of N-PPS and M-PPS

Treatments	L*	a*	b*
**N-PPS**	79.26 ± 0.71	-6.6 ± 0.12	7.84 ± 0.21
**M-PPS**	50.18 ± 0.63	-4.7 ± 0.12	6.44 ± 0.23

Table S2: Antifungal effect (% Radial growth inhibition) of *E. citriodora* essential oil

Concentration (µl/ml)	RGI (%)	
	P. griseofulvum	***A. niger***
Control	0.00 ± 0.00	0.00 ± 0.00
100	100.00 ± 0.00	100.00 ± 0.00
50	100.00 ± 0.00	100.00 ± 0.00
25	100.00 ± 0.00	100.00 ± 0.00
12.5	100.00 ± 0.00	100.00 ± 0.00
6.25	100.00 ± 0.00	100.00 ± 0.00
3.125	100.00 ± 0.00	100.00 ± 0.00
1.56	38.45 ± 4.18	73.78 ± 3.81
0.78	8.64 ± 5.67	13.80 ± 4.81

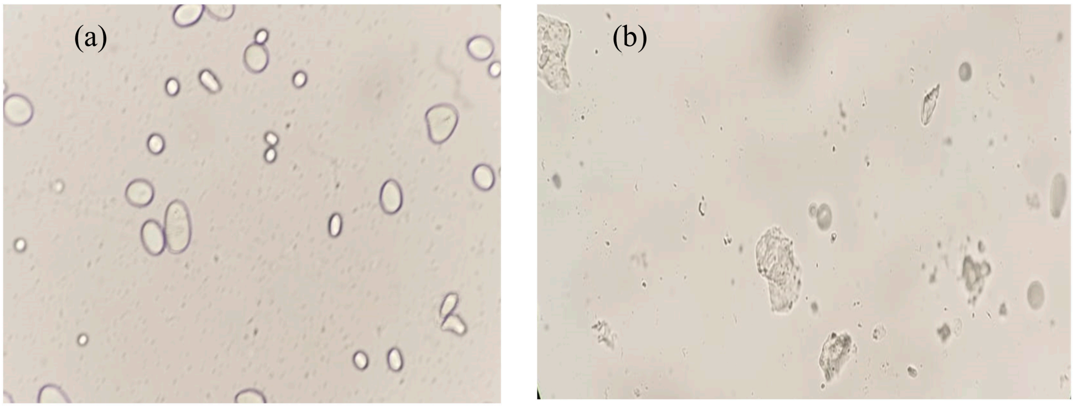

Figure S1. Morphological analysis of native (a) and modified (b) potato peel starch

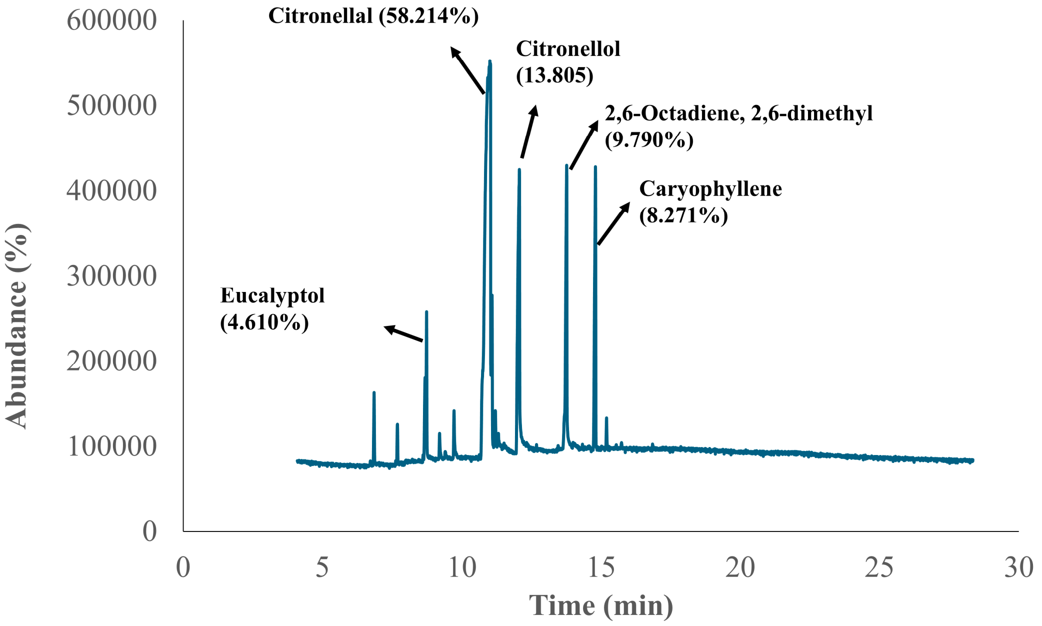

Figure S2. GCMS analysis of *E. citriodora* EO

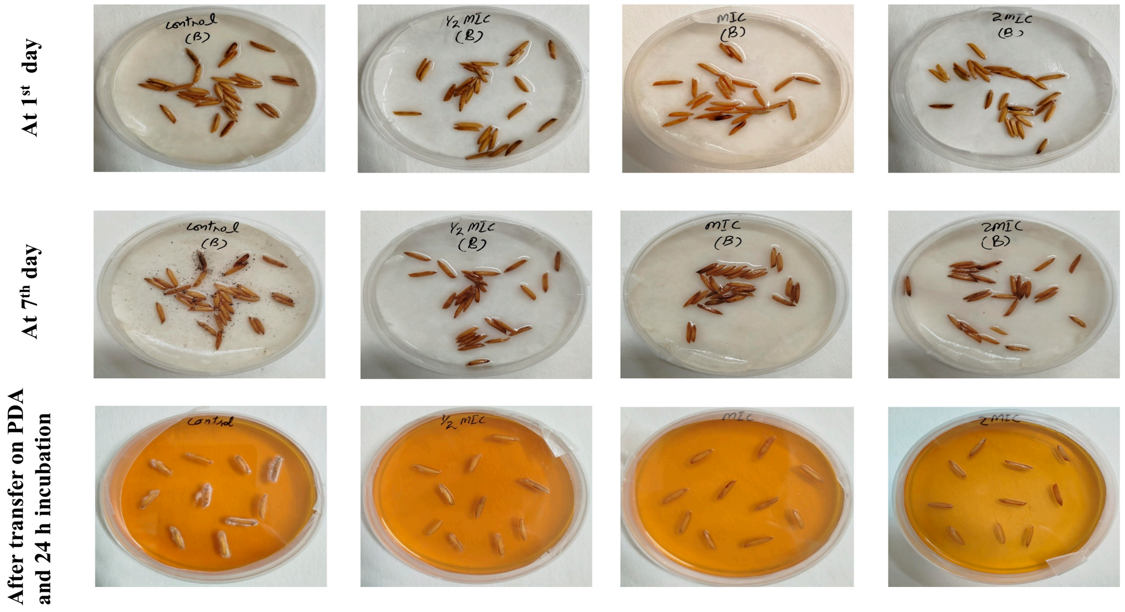

Figure S3. Antifungal effect of *E. citriodora* EO on rice grains inoculated with *A. niger*

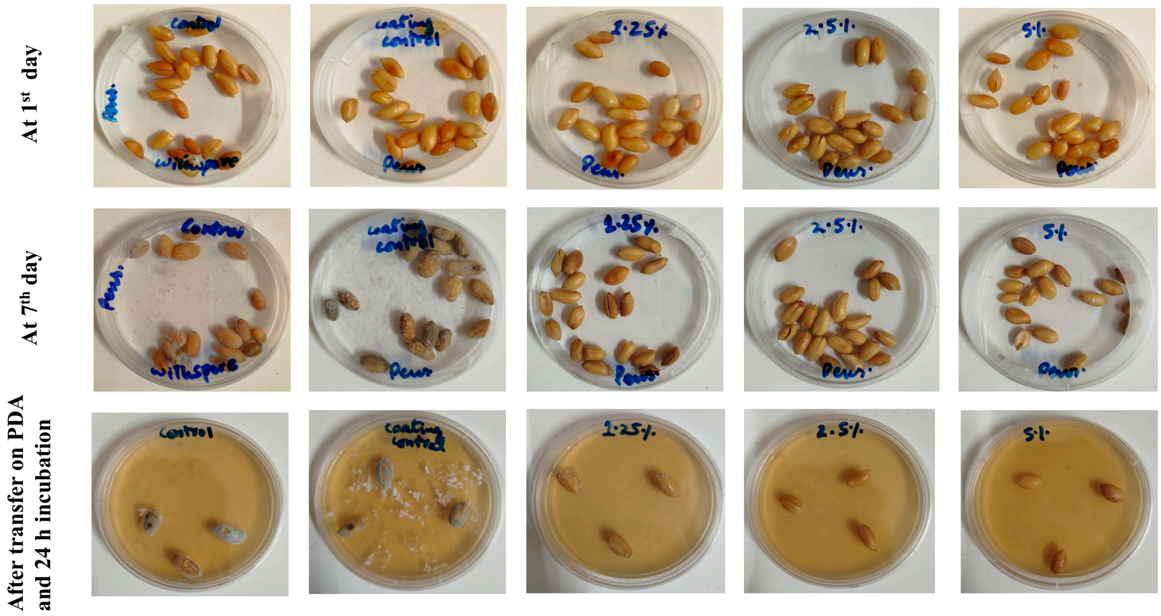

Figure S4. Antifungal effect of modified potato peel starch and *E. citriodora* based coatings on peanut kernels, inoculated with *P. griseofulvum*
